# Epidural versus intravenous fentanyl for postoperative analgesia following orthopedic surgery: randomized controlled trial

**DOI:** 10.1590/S1516-31802010000100002

**Published:** 2010-01-07

**Authors:** Marcelo Soares Privado, Adriana Machado Issy, Vera Lucia Lanchote, João Batista Santos Garcia, Rioko Kimiko Sakata

**Affiliations:** 1 MD, PhD. Anesthetist, Department of Anesthesia, Universidade Federal do Maranhão (UFMA), São Luis, Maranhão, Brazil.; 2 PhD. Assistant professor and pharmacologist, Department of Anesthesia, Universidade Federal de São Paulo — Escola Paulista de Medicina (Unifesp-EPM), São Paulo, Brazil.; 3 PhD. Titular professor and toxicologist, Department of Toxicology, Universidade de São Paulo (USP), Ribeirão Preto, São Paulo, Brazil.; 4 MD, PhD. Assistant professor and anesthetist, Department of Anesthesia, Universidade Federal do Maranhão (UFMA), São Luís, Maranhão, Brazil.; 5 MD, PhD. Associate professor, anesthetist and coordinator of the Pain Clinic, Department of Anesthesia, Universidade Federal de São Paulo — Escola Paulista de Medicina (Unifesp-EPM), São Paulo, Brazil

**Keywords:** Analgesia, Analgesia, epidural, Fentanyl, Pharmacology, Orthopedics, Analgesia, Analgesia epidural, Fentanila, Farmacologia, Ortopedia

## Abstract

**CONTEXT AND OBJECTIVE:**

Controversy exists regarding the site of action of fentanyl after epidural injection. The objective of this investigation was to compare the efficacy of epidural and intravenous fentanyl for orthopedic surgery.

**DESIGN AND SETTING:**

A randomized double-blind study was performed in Hospital São Paulo.

**METHODS:**

During the postoperative period, in the presence of pain, 29 patients were divided into two groups: group 1 (n = 14) received 100 µg of fentanyl epidurally and 2 ml of saline intravenously; group 2 (n = 15) received 5 ml of saline epidurally and 100 µg of fentanyl intravenously. The analgesic supplementation consisted of 40 mg of tenoxicam intravenously and, if necessary, 5 ml of 0.25% bupivacaine epidurally. Pain intensity was evaluated on a numerical scale and plasma concentrations of fentanyl were measured simultaneously.

**RESULTS:**

The percentage of patients who required supplementary analgesia with tenoxicam was lower in group 1 (71.4%) than in group 2 (100%): 95% confidence interval (CI) = 0.001-0.4360 (P = 0.001, Fisher’s exact test; relative risk, RR = 0.07). Epidural bupivacaine supplementation was also lower in group 1 (14.3%) than in group 2 (53.3%): 95% CI = 0.06-1.05 (P = 0.03, Fisher’s exact test; RR = 0.26). There was no difference in pain intensity on the numerical scale. Mean fentanyl plasma concentrations were similar in the two groups.

**CONCLUSION:**

Intravenous and epidural fentanyl appear to have similar efficacy for reducing pain according to the numerical scale, but supplementary analgesia was needed less frequently when epidural fentanyl was used.

**CLINICAL TRIAL REGISTRATION NUMBER:**

NCT00635986

## RESUMO

**CONTEXTO E OBJETIVO:**

Existem controvérsias sobre o local de ação do fentanil injetado por via peridural. O objetivo foi comparar a eficácia do fentanil peridural e do venoso em cirurgias ortopédicas.

**TIPO DE ESTUDO E LOCAL:**

Estudo aleatório, duplo-cego, realizado no Hospital São Paulo.

**MÉTODO:**

No pós-operatório, na presença de dor, 29 pacientes foram divididos em dois grupos: grupo 1 (n = 14) recebeu solução de 100 mcg de fentanil por via peridural e 2 ml de solução salina venosa; grupo 2 (n = 15), 5 ml de solução salina peridural e 100 µg de fentanil venoso. A complementação analgésica foi com 40 mg de tenoxicam venoso e, se necessário, 5 ml de bupivacaína 0.25%. A intensidade da dor foi avaliada pela escala numérica e a concentração plasmática do fentanil foi medida simultaneamente.

**RESULTADOS:**

A percentagem de pacientes que necessitaram de complementação analgésica com tenoxicam foi menor no grupo 1 (71.4% *versus* 100.0% grupo 2): intervalo de confiança, IC 95% = 0.001-0.4360 (P = 0.001, teste exato de Fisher; risco relativo, RR = 0.07). A complementação com bupivacaína peridural também foi menor no grupo 1 (14.3% *versus* 53.3% grupo 2): IC 95% = 0.06-1.05 (P = 0.03, teste exato de Fisher; RR = 0.26). Não houve diferença na intensidade da dor avaliada pela escala numérica. As concentrações plasmáticas do fentanil foram semelhantes nos dois grupos.

**CONCLUSÃO:**

A eficácia do fentanil venoso e peridural parece ser semelhante na redução da dor de acordo com a escala numérica, porém a frequência de analgesia suplementar foi menor com o uso do fentanil peridural.

## INTRODUCTION

The discovery that opioids act by activating receptors located in the dorsal horn of the spinal cord, and reports of intense and long-lasting pain relief after intrathecal injection of morphine^1^ have been important steps for postoperative analgesia.

Fentanyl is one of the most widely used opioids administered epidurally. The risk of respiratory depression is low and the incidence of side effects (itching, nausea, vomiting and urine retention) is lower than that observed with morphine.^2^ Controversy exists regarding the site of action of fentanyl after epidural injection. Some authors have suggested that its effect is directly mediated in the spinal cord, while others have believed that the main site of action is the supraspinal region, in view of its high liposolubility, with consequent absorption into the systemic circulation and distribution to the brain.^3^ Other investigators have suggested a combination of these two mechanisms to explain the action of epidural fentanyl.^4^ Thus, further studies are necessary to explain the sites of action of this drug when administered epidurally and also to determine whether there is any difference in the analgesic effect between applying the drug intravenously and epidurally.

## OBJECTIVE

To evaluate the efficacy of fentanyl (100 µg) administered by the epidural or intravenous routes after lower-limb orthopedic surgery, taking the primary outcome to be pain and the secondary outcome to be the requirement for supplementary analgesia during the postoperative period.

## METHODS

Twenty-nine consecutive patients admitted during a one-year period (from February 2005 to January 2006), of both genders and ranging in age from 18 to 65 years, were studied. They were all classified as presenting American Society of Anesthesiologists (ASA) physical status 1 or 2 and had been scheduled for orthopedic bone surgery on the lower limbs. The study was approved by the Ethics Committee of Hospital São Paulo, Medical School, Universidade Federal de São Paulo — Escola Paulista de Medicina (Unifesp-EPM), and written informed consent was obtained from all patients.

Patients with infection in the puncture area, those with coagulation disorders, pregnant patients, and patients using opioids were excluded from the study.

The study was conducted in a double-blind manner and each patient was randomly assigned to one of two groups. The process of randomization consisted of drawing lots for the procedures, which were kept in sealed envelopes. The draw was performed by a single physician who prepared the medication. Another physician was responsible for injection of the epidural and venous medication, while the researcher was responsible for evaluation of the patients. Both physicians and the patients were unaware of the group chosen by lot until the end of the study.

The medications were prepared in the same volumes and solution colors for the two groups. Thus, both the physician responsible for the procedure and the researcher were unaware of the group to which the patient belonged.

The epidural anesthesia consisted of 20 ml of 0.5% bupivacaine together with epinephrine (1:200,000), injected between L3-L4 and L4-L5 and supplemented with 5 ml of bupivacaine when necessary. After injection, an epidural catheter was inserted for postoperative analgesia. Sedation was achieved using 2-5 mg of midazolam.

During the postoperative period, when the patient complained of pain, the group 1 patients (n = 14) received 5 ml of a solution containing 100 µg of fentanyl diluted in saline epidurally and 2 ml of saline intravenously. The group 2 patients (n = 15) received 5 ml of saline epidurally and 2 ml of fentanyl (100 µg) intravenously. Thus, the patients of both groups received both an intravenous and an epidural solution, thus ensuring double-blind study conditions, i.e. group 1: epidural fentanyl + intravenous saline (placebo); group 2: epidural saline (placebo) + intravenous fentanyl.

If supplementation was necessary, 40 mg of tenoxicam was administered intravenously, and if the patient continued to complain of pain, 5 ml of 0.25% bupivacaine was injected.

The pain intensity was evaluated 30 minutes, two hours and four hours after injection of fentanyl, on a scale ranging from 0 (no pain) to ten (very intense pain).^5,6^ On the same occasions, venous blood samples were collected for measurement of plasma fentanyl concentrations. The blood samples were immediately centrifuged and the plasma was separated and stored at -70 °C until the time for spectrochromatography analysis. The quantification limit of the method was 0.05 ng/ml of plasma.

Thirty patients were originally selected (15 per group), but one patient in group 1 was excluded from the study because blood entered the epidural catheter.

For statistical analysis, the distribution of frequency data was analyzed by means of the chi-square test using the GraphPad InStat software. Two-way repeated-measurement analysis of variance (ANOVA) was used to compare the samples, repeated over time. Student’s t test, Fisher’s exact test, and Mann Whitney test were also applied.

Our hypothesis was that intravenous fentanyl was associated with a response rate of 30%. Assuming that epidural fentanyl would have a response rate of 80% improvement in pain response (primary outcome) and considering a power of 95% (beta) and P = 0.05% (alpha), the sample size would be 15.54.

## RESULTS

There were no significant differences between the groups with regard to gender, age, weight, height or body mass index (Table 1). In addition, no significant difference in the duration of surgery was observed between the groups (137 minutes for group 1 and 135 minutes for group 2). The interval between the beginning of anesthesia and the administration of epidural fentanyl (group 1) was 380 ± 101 minutes, and the interval between the beginning of anesthesia and the administration of intravenous fentanyl (group 2) was 331 ± 83 minutes, with no significant difference between the groups (Student’s t test, P = 0.17).

**Table 1.** Demographic data on the patients

	Gender (F, M)	Age (years)	Weight (kg)	Height (cm)	BMI (kg/m^2^)
Group 1 (n = 14)	3F; 11 M	35.28 ± 15.68	71.14 ± 11.79	170.93 ± 8.27	24.42 ± 4.12
Group 2 (n = 15)	5F; 10 M	41.26 ± 13.24	74.73 ± 9.25	167.87 ± 8.32	26.52 ± 2.46
P	0.6817	0.2762^1^	0.3632^1^	0.5015^1^	0.1628^1^

Group 1 = epidural fentanyl; Group 2 = intravenous fentanyl; P = statistical significance, ≤ 0.05; M = male; F = female; BMI = body mass index; p = Fisher’s exact test; P^1^ = Student’s t test; values expressed as mean ± standard deviation.

The percentage of patients who required supplementary analgesia with tenoxicam was lower in group 1 (71.4%) than in group 2 (100.0%): 95% confidence interval (CI) = 0.001-0.4360 (Fisher’s exact test, P = 0.04; risk relative, RR = 0.07). Epidural bupivacaine supplementation was also lower in group 1 (14.3%) than in group 2 (53.3%): 95% CI = 0.06-1.05 (Fisher’s exact test, P = 0.05; RR = 0.26). The first analgesic supplementation was necessary after 125.7 ± 57.6 minutes in group 1 and after 98.3 ± 49.5 minutes in group 2, without any significant difference (Mann-Whitney test, P = 0.267). No significant difference in pain intensity evaluated on the numerical scale was observed between the groups (Table 2).

**Table 2.** Pain intensity at each evaluation time (mean ± standard error of the mean)

	Time (minutes)			P^1^
	T30	T120	T240	
Group 1 (n = 14)	0.35 ± 0.22	2.07 ± 0.86	2.35 ± 0.65	0.0112
95% CI	- 0.13 - 0.84	0.21 - 3.93	0.95 - 3.76	
Group 2 (n = 15)	0.86 ± 0.43	3.60 ± 0.70	2.46 ± 0.80	0.0101
95% CI	- 0.06 - 1.80	2.09 - 5.10	0.73 - 4.20	
P^2^	0.7013	0.0843	0.8794	

Group 1 = epidural fentanyl; Group 2 = intravenous fentanyl; T_30_; T_120_; T_240_ = times (min) after the administration of fentanyl; 95% CI = 95% confidence interval; P^1^ = analysis of variance (ANOVA); P^2^ = Student’s t test.

The mean plasma fentanyl concentrations (ng/ml) were similar in the two groups (ANOVA, P = 0.67; Table 3). Tables 4, 5 and 6 show the cumulative numbers and percentages of patients who had required supplementary analgesics up to the time of each evaluation (T_0_, T_30_, T_120_ and T_240_), as well as the mean pain intensity and plasma fentanyl concentrations at these times.

**Table 3.** Fentanyl plasma concentrations (ng/ml)

	Time (min)			P^1^
	T30	T120	T240	
Group 1 (n = 14)	0.21 ± 0.06	0.23 ± 0.15	0.29 ± 0.41	0.6642
95% CI	0.17 - 0.24	0.15 - 0.32	0.06 - 0.53	
Group 2 (n = 15)	0.31 ± 0.16	0.21 ± 0.10	0.16 ± 0.08	0.0008
95% CI	0.22 - 0.39	0.15 - 0.26	0.11 - 0.21	
P^2^	0.0734	0.9304	0.3480	

Group 1 = epidural fentanyl; Group 2 = intravenous fentanyl; T_30_; T_120_; T_240_: times (min) after the administration of fentanyl; 95% CI = 95% confidence interval; P^1^ = analysis of variance (ANOVA); P^2^ = Student’s t test.

**Table 4.** Cumulative number of applications of supplementary analgesic, pain intensity and plasma fentanyl concentrations among the patients, 30 minutes after administration of epidural or intravenous fentanyl (T_30_)

	None			Tenoxicam			Bupivacaine		
	G1	G2	P	G1	G2	P	G1	G2	P
N (%)	13 (93)	15 (100)	0.4828*	1 (7)	0 (0)	0.4828	0 (0)	0 (0)	–
NS	0.2	0.5	0.5186†	3.0	–	–	–	–	–
C (ng/ml)	0.4	0.3	0.6530†	0.3	–	–	–	–	–

NS = numerical scale; C = plasma fentanyl concentration; T_30_ = 30 minutes after administration of epidural or intravenous fentanyl;

* Fisher’s exact test;

† Mann-Whitney test

**Table 5.** Cumulative number of applications of supplementary analgesic, pain intensity and plasma fentanyl concentrations among the patients, 120 minutes after administration of epidural or intravenous fentanyl (T_120_)

	None			Tenoxicam			Bupivacaine		
	G1	G2	P	G1	G2	P	G1	G2	P
N (%)	8 (57)	3 (20)	0.0704*	6 (43)	12 (80)	0.0604*	1 (7)	3 (20)	0.5977*
NS	1.5	2.6	0.5196†	3.0	4.0	0.9999†	10	0.5	–
C (ng/ml)	0.2	0.2	0.3727†	0.2	0.2	0.4936†	0.26	0.3	–

NS = numerical scale; C = plasma fentanyl concentration; T_20_ = 120 minutes after administration of epidural or intravenous fentanyl

* Fisher’s exact test

† Mann-Whitney test

**Table 6.** Cumulative number of applications of supplementary analgesic, pain intensity and plasma fentanyl concentrations among the patients, 240 minutes after administration of epidural or intravenous fentanyl (T_240_)

	None			Tenoxicam			Bupivacaine		
	G1	G2	P	G1	G2	P	G1	G2	P
N (%)	10 (71)	8 (53)	0.4497*	3 (21)	3 (20)	1.0000*	1 (7)	5 (33)	0.1686*
NS	2.4	3.8	0.6965†	1.3	4.0	0.7000†	2.5	0.5	–
C (ng/ml)	0.3	0.2	0.1728†	0.2	0.1	1.0000†	0.2	0.3	–

NS = numerical scale; C = plasma fentanyl concentration; T_240_ = 240 minutes after administration of epidural or intravenous fentanyl

* Fisher’s exact test

† Mann-Whitney test

In group 1, at T_30_, the mean pain intensity among the patients who did not require supplementary analgesics (n = 13; 93%) was 0.2 and the plasma fentanyl concentration was 0.20 ng/ml. The single patient (7%) who received tenoxicam had a pain intensity score of 3 and a plasma fentanyl concentration of 0.27 ng/ml. At T_120_, the mean pain intensity among the patients who still had not required analgesic supplements (n = 7; 50%) was 0.3 and the plasma fentanyl concentration was 0.23 ng/ml. For the patients who received tenoxicam (n = 7; 50%), the pain intensity score was 3.9 and the mean plasma fentanyl concentration was 0.23 ng/ml. The single patient (7%) who received bupivacaine presented a pain intensity score of 10 and a plasma fentanyl concentration of 0.26 ng/ml. At T_240_, the mean pain intensity among the patients who had not required analgesic supplements until that time (n = 4; 29%) was 2.2 and the plasma fentanyl concentration was 0.55 ng/ml. The pain intensity score of the patients who received tenoxicam (n = 10; 71%) was 2.4 and the plasma fentanyl concentration was 0.19 ng/ml. For the patients who received bupivacaine (n = 2; 14%), the pain intensity score was 2.5 and the plasma fentanyl concentration was 0.16 ng/ml.

In group 2, at T_30_, the mean pain intensity among the patients who did not require tenoxicam supplementation (n = 15; 100%) was 0.9 and the plasma fentanyl concentration was 0.31 ng/ml. At T_120_, the pain intensity among the patients who did not require analgesic supplements (n = 3; 20%) was 3.4 and the plasma fentanyl concentration was 0.21 ng/ml. The pain intensity among the patients who received tenoxicam (n = 12; 80%) was 4 and the plasma fentanyl concentration was 0.20 ng/ml. For the patients who received bupivacaine (n = 3; 20%), the pain intensity score was 2 and the plasma fentanyl concentration was 0.24 ng/ml. At T_240_, all the patients had received tenoxicam supplementation, and these patients (n = 15; 100%) presented a pain intensity score of 2.5 and a plasma fentanyl concentration of 0.16 ng/ml. The pain intensity among the patients who had received bupivacaine up to that time (n = 8; 54%) was 0.6 and the plasma fentanyl concentration was 0.19 ng/ml.

The side effects observed were nausea (group 1: one patient; group 2: one patient), vomiting (group 2: one patient), and urine retention (group 2: two patients).

## DISCUSSION

Several investigators^7-20^ have evaluated the mechanism of action of fentanyl administered epidurally. Although many studies^3,9-12,15,17^ have concluded that the mechanism of action is associated with systemic absorption of the opioid into the circulation, followed by a supraspinal effect, many investigators continue to use fentanyl as a continuous infusion. According to the literature,^2,9^ with continuous infusion, the higher plasma concentration of fentanyl promotes supraspinal action. In these studies, the plasma fentanyl concentrations were around 0.63 ng/ml for patients to maintain pain relief. We used a single bolus of epidural fentanyl.

Although methods for measuring plasma fentanyl concentrations are available, they do not reflect the degree of activation of opioid receptors by the drug.^10^ Studies evaluating the site of action of epidural fentanyl have usually focused on the plasma concentration of the drug, its analgesic effect and the need for supplementary analgesia. However, a greater challenge would be to study the binding of this drug to its receptor.

The need for supplementary analgesia was greater in group 2 than in group 1. This finding suggests that epidural fentanyl is more efficient than intravenous administration of the drug, as also reported in another study.^12^ Although the statistical analysis did not show any difference in the length of time before the first request for supplementary analgesia, analgesic supplementation occurred earlier in patients who had received intravenous fentanyl, thus suggesting that epidural fentanyl has a longer-lasting effect. Another interesting point was that only two patients needed both types of analgesic supplements after epidural fentanyl while, after intravenous fentanyl, eight patients required both supplements.

The intensity of pain was similar in the two groups, and this has also been reported in other studies.^11,13,21^ This finding shows that both intravenous and epidural fentanyl promoted good pain relief within the first 30 minutes. However, after this period, part of the analgesic effect was mediated by supplementation. Other investigators also observed that, after the first hour, the pain was less intense after epidural fentanyl than after intravenous administration, despite the similar or even lower plasma concentrations.

A correlation between plasma opioid concentration and pain relief has been reported.^22-24^ The minimum plasma fentanyl concentration for postoperative pain relief was 0.63 ng/ml.^2^

The plasma fentanyl concentrations observed at different times were similar to those reported in other studies.^3,11,15,21^ Some investigators found that the plasma concentration of fentanyl was much lower after epidural injection than after intravenous administration.^12,16^

In the present study, only two patients in group 1 and one patient in group 2 presented a fentanyl concentration of 0.63 ng/ml during the period analyzed.

Another important point was that, in group 1, most patients presented good pain relief at T_30_, with plasma fentanyl concentrations lower than 0.63 ng/ml in the absence of any supplementary analgesia. In addition, many group 2 patients showed good pain relief despite a plasma fentanyl concentration below the minimum required for analgesia, although the number of patients was smaller than in group 1. At T_120_, many group 1 patients still presented good pain relief with plasma fentanyl levels less than 0.63 ng/ml and did not require supplementary analgesics. In group 2, few patients presented adequate pain relief and all of them requested supplementary analgesia. After four hours, some group 1 patients continued to present good pain relief, although they did not receive any analgesic supplement, and their plasma fentanyl concentration was less than 0.63 ng/ml. Four hours after epidural injection, the plasma fentanyl concentration was higher than after two hours, probably because it was spreading out from the distribution sites of the drug.

The better analgesic effect of epidural fentanyl observed in the present study, despite the lower plasma concentration of the drug, suggests that there is an effect caused by spinal action, in addition to the systemic action.

The side effects associated with fentanyl were nausea and vomiting. Urine retention, an adverse effect more frequently observed after epidural opioids, was observed in the intravenous group. This effect was probably associated with the epidural bupivacaine used for anesthesia.

## CONCLUSION

Among patients undergoing lower-limb orthopedic surgery, intravenous and epidural fentanyl appear to have similar efficacy for reducing pain according to the numerical scale, but supplementary analgesia was needed less frequently when epidural fentanyl was used.
